# The Uses of Pure Tannin

**Published:** 1845-02

**Authors:** Robert Druitt


*The uses of Pure Tannin.* By Robert Druitt, Esq.—In any case in which a vegetable astringent is indicated, Mr. Druitt believes that the tannin ought to have the preference. A simple solution of it, in distilled water, he says, is much more easily and quickly prepared, as well as much more elegant, than the ordinary decoctions or infusions of oak-bark, catechu, &c.; moreover, it may be prepared of uniform strength, and free from foreign inert matter, and is not liable to decompose quickly; in fact, it has all the advantage which the other simple vegetable principles have over crude preparations from the herbs or extracts in which they are contained.

The cases in which Mr. D. has employed it, are sore nipples, excoriations about the anus and scrotum, piles, leucorrhœa, atonic phagedenic sores, tooth ache, aphthous sores in the mouth, severe salivation and relaxed sore throat.

For sore nipples especially, Mr. D. has found it "invaluable." Every accoucheur knows what a source of wretchedness and illness these are to the young mother, and how difficult it often is to find a decisive remedy; but Mr. D. has never been disappointed in the use of tannin, except once, in a neglected case, with deep irratable cracks, for which it was necessary to use the lunar caustic. The form in which he employed it, is a solution of five grains in an ounce of distilled water; this is applied to the nipple on lint, covered with oiled silk.

For the itching excoriations about the anus and scrotum, which so much infest old men, he has used it with benefit, but prefers lemon juice as a local application. For piles, with mucous discharge, he has also found it of use, but he cannot say much on this point from his own experience.

"In one or two cases of lingering atonic phagedena," says Mr. D., "I have found it of some service, sprinkled thickly on the sore; but more particularly so in those aphthous ulcers which sometimes occur in the mouths of adults, from acidity of the stomach, and congestion of the liver. I may say that I believe it the best possible remedy for severe salivation, and for all cases of relaxed sore-throats attended with superabundance of mucus. It coagulates the mucus and enables the patient to get rid of it easily. Of course I do not use it to the exclusion of constitutional remedies; but of all the local means of making the mouth *comfortable*, I believe it to be the best.

"But of all the cases for which it is adapted, that common troublesome complaint, tooth-ache, is that in which I believe it is most to be depended on. For this piece of useful knowledge I am indebted to my friend Mr. Tomes, and I have tested it by ample personal experience. It will be found, as Mr. Tomes told me, that the gum around a carious tooth is in a spongy, flabby condition; a little piece of it, perhaps, growing into the cavity. The ache, too, is often quite as much in the gum, as in the tooth itself. But, be this as it may, when the tooth aches let the patient wash out the mouth thoroughly with a solution of carbonate of soda in warm water; let the gum around the tooth, or between it and its neighbor, be scarafied with a *fine* lancet; then let a little bit of cotton wool, imbued with a solution of a scruple of tannin, and five grains of mastich, in two drachms of æther, be put into the cavity, and if the ache is to be cured at all, this plan will put an end to it, in nine cases out of ten. I think that practitioners are to blame in not paying more attention to the cure of tooth-ache; I am convinced, that in most cases, it is as curable as a colic or a pleurisy; the chief points being to open the bowels, and put the secretions of the mouth in a healthy state, and to apply some gentle astringent and defensative to the diseased tooth, till it is capable of being stopped by some metallic substance. I say emphatically a *fine* lancet, because the coarse, round, blunted tools that are generally sold under the name of gum-lancets, only bruise the gum, and cause horrible pain. The lancet which I use is sickle-shaped, cutting on both edges and finely ground; and if guarded with the middle finger of the right hand, it may be used in the case of the most unruly children, without any possible ill result."—Prov. Med. and Surg. Jour., Oct. 9, 1844.

